# Nanofabrication and Demonstration of a Direct‐Write Microevaporator

**DOI:** 10.1002/smsc.202300121

**Published:** 2023-12-22

**Authors:** Xella Doi, Pavani Vamsi Krishna Nittala, Brian Fu, Kyaw Zin Latt, Suryakant Mishra, Luke Silverman, Linus Woodard, Ralu Divan, Supratik Guha

**Affiliations:** ^1^ Pritzker School of Molecular Engineering University of Chicago Chicago IL 60637 USA; ^2^ Materials Science Division Argonne National Laboratory Lemont IL 60439 USA; ^3^ Center for Nanoscale Materials Argonne National Laboratory Lemont IL 60439 USA

**Keywords:** direct write, heterogeneous integration, high-aspect-ratio etch, nanofabrication, physical vapor deposition

## Abstract

Direct‐write vapor deposition is a new technique that would enable one‐step 3D maskless nanofabrication on a variety of substrates. A novel silicon chip‐based microevaporator is developed that allows evaporant to exit through 2000–300 nm nozzles while held at distances comparable to the nozzle diameter from the substrate by a three‐axis nanopositioning stage in vacuum. This results in a localized deposition on the substrate, which may be scanned relative to the substrate to produce direct‐write patterns. The performance of the microevaporator is tested by creating localized depositions of various materials and the line‐writing potential is demonstrated. The relationship between linewidth and source‐to‐substrate distance is investigated by the application of Knudsen's cosine law and Monte‐Carlo simulations, and then utilized to approximate the source‐to‐substrate distance from performed depositions.

## Graphical abstract

A silicon‐based microevaporator is developed for maskless patterning of thin films by direct‐write vapor deposition. It allows material to exit via 2 to 0.3 μm diameter nozzles to deposit on a substrate. Used with a scanning stage, writing of 1 μm diameter dots and 2 μm wide lines is demonstrated. (The table of contents figure is an artistic rendition using stitched microscopy images and graphics).

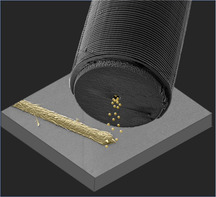

## 1 Introduction

There is growing interest today in the microelectronics industry for creating 3D chip architectures and in the heterogeneous integration (HI) of components.^[^ ^1^, ^2^, ^3^, ^4^ ^]^ This has led to the need for alternatives to 2D lithography‐based nanofabrication which was originally developed to meet the needs of planar chip technologies.^[^ ^1^, ^2^, ^5^, ^6^ ^]^ Recently, there has been interest in additive “direct write” approaches^[^ ^7^, ^8^, ^9^, ^10^, ^11^, ^12^, ^13^, ^14^, ^15^, ^16^, ^17^, ^18^, ^19^, ^20^, ^21^, ^22^, ^23^, ^24^, ^25^, ^26^, ^27^ ^]^ as an alternative method of nanofabrication with two significant opportunities for their utilization.

The first opportunity is in HI: creating dense interconnects between many chiplets with diverse functions and geometries that are integrated on large panels or packages, often organic substrates.^[^ ^3^, ^28^, ^29^ ^]^ Chiplet integration addresses the needs for energy‐efficient computing and the need for fast access to lots of memory.^[^ ^3^, ^29^ ^]^ The area of a single chip is ultimately limited by the reticle size during lithography, hence the integration of multiple chiplets is necessary. There is an opportunity in HI for a monolithic, preferably 3D compatible, fabrication process for creating interconnects and associated passive device components (such as inductors and capacitors) on a panel.^[^ ^3^, ^30^ ^]^ The reticle size limitation and need for diverse substrates represent an opportunity for new direct‐write methods. The specifications for direct‐write methods for this application would be resolutions from the nanoscale (≈100 nm) to micron range, high deposition speeds, ability to deposit ultrapure metals and dielectrics, and 3D compatibility.

The second opportunity for direct write is in augmenting conventional lithography to create features in the front end or back end of a chip that are more amenable to 3D structures. This may require feature size control in the 1–10 nm range. The creation of modern 3D device architectures by traditional lithography‐based nanofabrication requires many process steps. Additive manufacturing via direct‐write methods is inherently 3D, so it may overcome these limitations and simplify processing.

“Direct write”‐based additive manufacturing on the macroscopic scale is widely used for prototyping and commercial products.^[^ ^31^ ^]^ In nanofabrication, focused ion beam (FIB)‐ and focused electron beam‐induced depositions have been available for direct writing at the nanoscale, and are able to perform both material addition and subtraction,^[^ ^32^ ^]^ and features of sub‐20 nm dimensions have been demonstrated.^[^ ^33^ ^]^ However, challenges remain due to scalability, beam damage and contamination, and compatibility with insulating substrates. Direct‐write research has also included methods utilizing liquid‐phase precursors and methods including inkjet printing, extrusion, and photocuring.^[^ ^1^ ^]^ Many of these approaches face the challenge of developing unique inks or precursors suitable for the deposition of ultrahigh purity materials with high uniformity. Gas‐phase or physical evaporation‐based direct‐write technologies offer the potential of high purity and perhaps the potential for extreme nanoscale depositions.^[^ ^5^, ^7^, ^8^, ^12^, ^27^ ^]^ There is a need for a physical vapor deposition (PVD)‐ or chemical vapor deposition (CVD)‐based technique that can provide the combinations of high growth rate, resolutions in the sub‐10 nm to tens of micrometers range (depending upon application), and ability to deposit metals and dielectrics.

**Figure 1:**
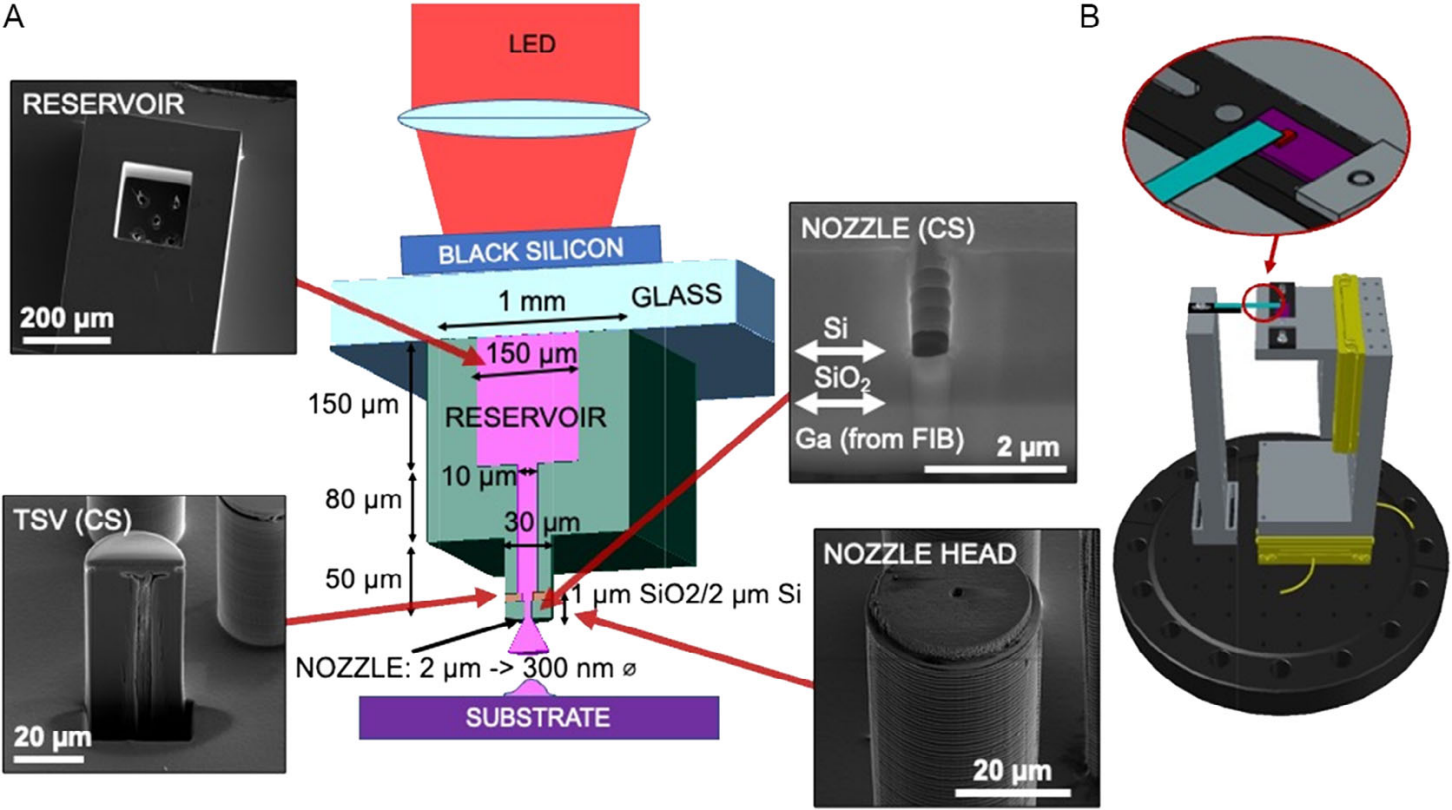
System overview. The microevaporator fabricated from silicon is held in place above substrate and heated to cause the evaporant to exit and deposit on substrate. A) System overview sketch with important features highlighted and shown on the device by scanning electron microscopy (SEM). Light from light‐emitting diode (LED) (red) is focused on the black silicon chip (dark blue), which conducts heat through the glass slide (light blue) to the microevaporator (green), resulting in heating of material (pink) in the reservoir to evaporate and travel via a thru silicon via (TSV) which terminates in a 2 μm to 300 nm nozzle. The nozzle is held above a substrate at distances of ≈20 μm to 500 nm such that the evaporant lands on the substrate (purple) to create a localized thin film deposition. The nozzle is centered on a 30 μm across nozzle head which allows for precise positioning and reduced thermal interaction between the microevaporator and substrate. B) Illustration of full system on scanning stage. The glass slide (blue) containing microevaporator (red) is held fixed above the substrate (purple) which can be scanned in 3D by the nanopositioning stages (yellow).

In this work, we have asked the question: can one miniaturize a physical vapor evaporator by building it on a silicon chip using microfabrication, and then scan it across a substrate to create a direct‐write process? We have developed a new gas‐phase direct‐write system, illustrated in **Figure** **1**a, which utilizes physical vapor deposition from a silicon‐based “microevaporator,” which is fabricated using scalable semiconductor processes. Heating of the entire microevaporator body during use prevents nozzle clogging so that a nozzle may be used indefinitely without cleaning (this is a “hot” nozzle).

Herein, we will introduce the design and fabrication of the microevaporator, as well as demonstrate a route to fabricating nozzle diameters as small as 30 nm. Microevaporator loading, deployment, and heating are discussed along with initial demonstrations of deposition capability. Localized thin film depositions grown by this method are compared with direct simulation Monte Carlo (DSMC) simulations and calculations based on Knudsen's cosine equation to use the expected shape of deposited material to approximate the source‐to‐substrate distance during deposition.

## 2 Experimental Results and Discussion

### 2.1 The Direct‐Write System: Design Considerations

The microevaporator device (Figure 1a) consists of a 150 μm across evaporant reservoir which is connected to a 10 μm diameter TSV which terminates in a 2000–300 nm diameter nozzle that is used to emit evaporant in a localized area. By integrating the vapor reservoir and nanonozzles onto a single 1 mm × 2 mm chip, we create a simple, self‐contained evaporator for performing the direct‐write vapor deposition. The microevaporator is used for PVD by filling the reservoir with solid material and sealing, and then heating the entire device to cause material to evaporate and exit via the nozzle. Figure 1a illustrates the microevaporator use. Note that the solid evaporant material has a grain size much larger than the size of the nozzles, so the material is not able to exit the nozzles in solid form and thus remains contained inside the microevaporator until it is heated to evaporate. Coupled with a three‐axis nanopositioning stage, as shown in Figure 1b, the microevaporator's nozzle is held at distances comparable to the nozzle diameter from the substrate surface to create localized depositions and scanned relative to the substrate to write patterns. The substrate is placed on a copper heatsink for thermal stability and can be further cooled by a liquid nitrogen cold finger. The entire system is housed in a vacuum chamber (10^−7 ^kPa base pressure) that is mounted on a pneumatic vibration isolation table.

In designing the system presented in this article, we identified several factors which influence the writing properties: nozzle size, approach distance, and influences of thermal interactions. In the following, we address all three of these aspects with the goal of minimizing the achievable linewidth of the deposition. To minimize nozzle size, we developed a nanofabrication process based on electron beam lithography (EBL), time‐multiplexed “Bosch” etching,^[^ ^34^ ^]^ and a nozzle diameter trimming approach with which we have been able to fabricate nozzles with diameters down to 30 nm in diameter on blank wafers. In the current article, the minimum nozzle diameter which has been integrated onto a full microevaporator device and tested is 300 nm.

To minimize approach distance, the microevaporator geometry is chosen such that the nozzle placement on the 30 μm diameter nozzle head (see Figure 1a, nozzle head) allows for close approach even with some angular mismatch between source and substrate. A nanopositioning stage and laser alignment technique is also implemented with the goal of minimizing approach distance.

The nozzle head geometry also reduces the thermal interaction between source and substrate because it minimizes the area of the microevaporator which is within the “near‐field” distance for thermal coupling.^[^ ^35^ ^]^ The thermal interactions in the system are dependent on the source‐to‐substrate distance and the use of temperature measurements is proposed as a method for controlling the source‐to‐substrate distance.

### 2.2 Microevaporator and Nozzle Fabrication

**Figure 2:**
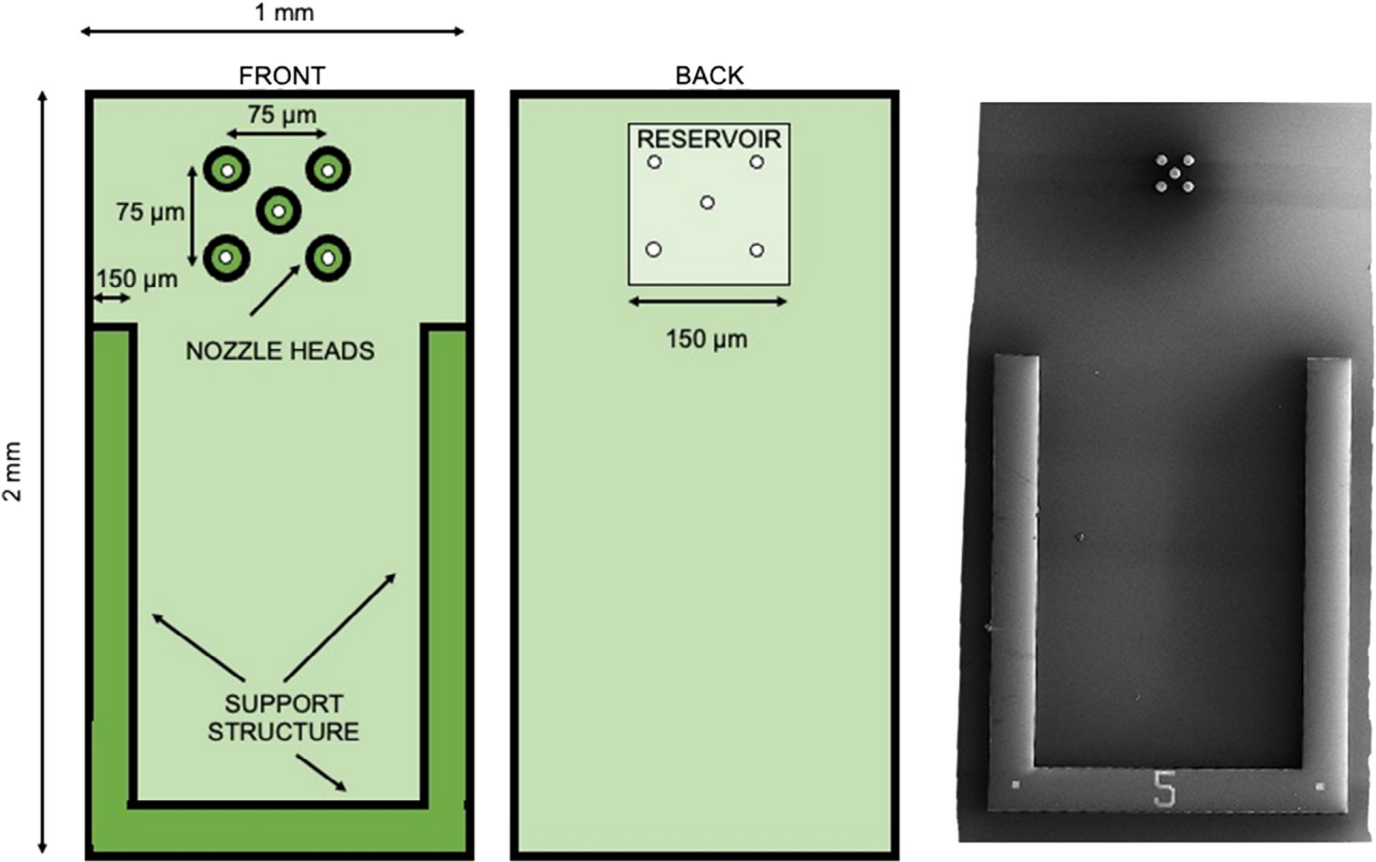
Overview of a full microevaporator device design. Illustration of full microevaporator body (not to scale) with relevant dimensions labelled and SEM of frontside of fabricated device. On the frontside, the nozzle heads and support structure are 50 μm tall. A single nozzle (white) is centered on each of the five nozzle heads. On the reverse side, the reservoir is 150 μm deep and feeds in to the 10 μm diameter TSVs that terminate in nozzles on the frontside. The frontside of this device has a support structure to reduce risk of damage to the nozzle heads.

The overall design of the microevaporator device can be seen in the schematic of **Figure** **2**. The basic geometry consists of a reservoir for evaporant material that is dispersed through a nozzle at the end of a pillar‐shaped raised nozzle head (Figure 1a). In practice, the layout of the overall device can contain multiple nozzle heads fed from the same reservoir, each containing its own nozzle. Alternately, multiple nozzle openings may be placed on a single nozzle head. In the current design, we fabricate five nozzle heads, each with a single nozzle centered on its face, as shown in Figure 2. The device also contains a support structure which helps avoid damaging the nozzle heads during fabrication and handling; however, this support structure cancels some of the heat transfer benefits of the nozzle head structure as well as increases the effective plane which is presented to the substrate surface, making the minimum achievable nozzle‐to‐substrate distance sensitive to discrepancies in tilt. The microevaporator can be fabricated without the support structure with no change to the overall process flow besides deletion of the structure from the relevant lithography step.

**Figure 3:**
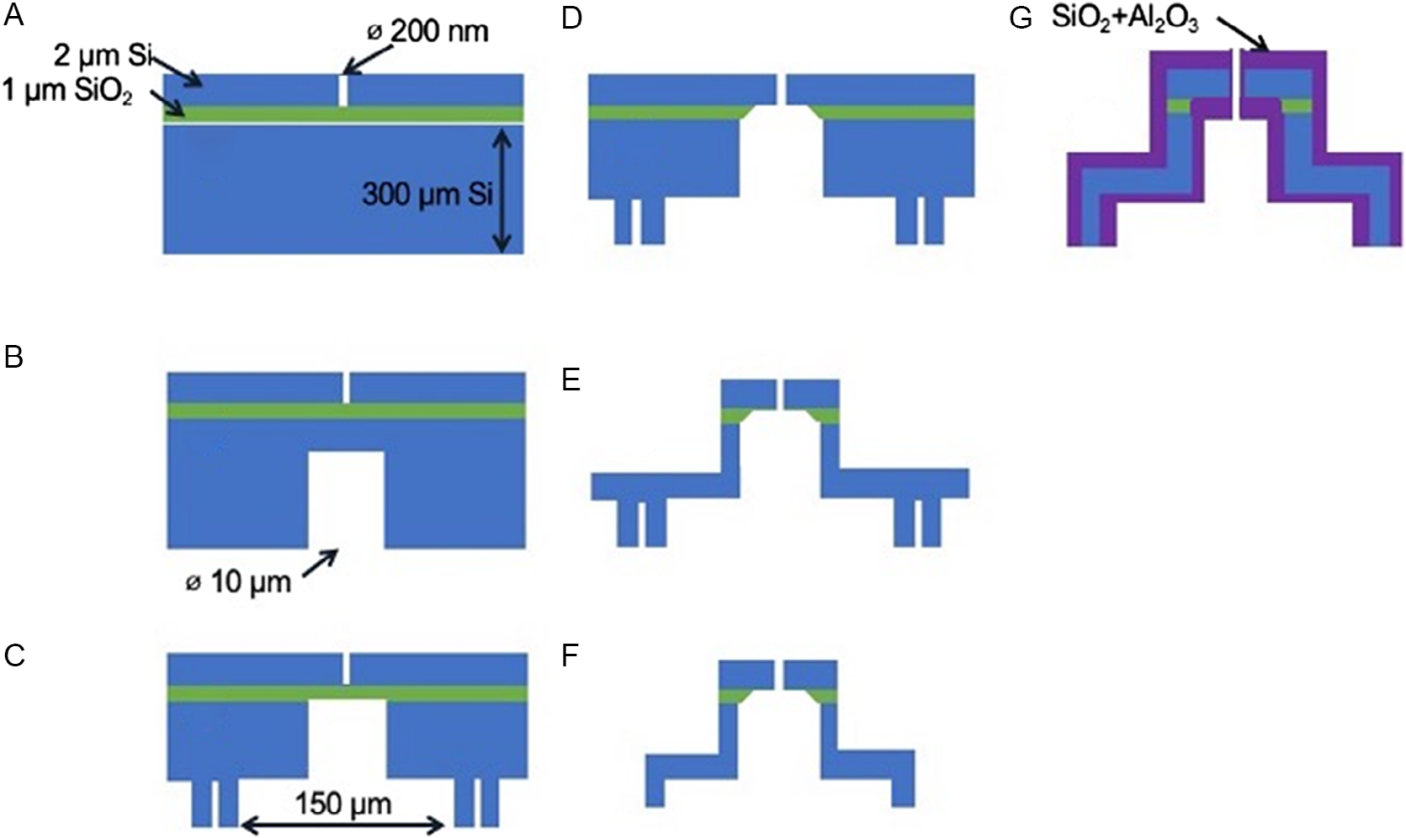
Illustration of process flow for fabrication of microevaporator. A) EBL and Bosch etch to define nozzle and alignment marks on the frontside. B) Optical lithography and Bosch etch on the reverse side to start TSV. C) Optical lithography and Bosch etch on the reverse side to define reservoir and cleave lines while further etching the TSV to touchdown on oxide. D) Oxide etch by RIE from the reverse side to clear oxide from nozzle opening. E) Optical lithography and Bosch etch through the top silicon, then RIE to etch through the oxide layer, and final Bosch etch to define the nozzle head structure. F) Manual die singulation along the etched cleave lines. G) Nozzle diameter trimming by conformal deposition of materials (plasma‐enhanced chemical vapor deposition [PECVD] SiO_2_ and atomic layer deposition [ALD] Al_2_O_3_).

We fabricated the microevaporator from a < 100> silicon‐on‐insulator wafer with a 2 μm thick top silicon layer, 1 μm thick SiO_2_ (“oxide”) layer, and a 300 μm thick handle layer. The process consists of over 120 individual steps grouped into seven major steps, which involve defining the nozzles by EBL (**Figure** **3**a **)**, etching of high‐aspect‐ratio features by reactive ion etch (RIE) using the Bosch etch technique^[^ ^36^ ^]^ (Figure 3a,b,c,e**)** from both the front and reverse sides. The fabrication process flow is summarized in Figure 3. The first step, defining the nozzle (Figure 3a), is the only step that requires EBL, for the rest of the process lithography is performed by optical lithography. The oxide serves as an etch stop for the Bosch process and must be removed by a separate dry etch process (Figure 3d) because a wet etch results in loss of the top membrane containing the nozzle opening. A detailed description of all process steps can be found in the Supporting Information, Section S1. A discussion of the challenges associated with high‐aspect‐ratio etch and the recipes used in this work is provided in Supporting Information Section S2 and S3, respectively.

#### 2.2.1 Nozzle Diameter Trimming

The resist thickness required to survive the Bosch etch through the 2 μm‐thick top silicon layer which defines the nozzle limits the smallest nozzle size defined by EBL to around 100 nm, so alternative methods are required to further reduce the nozzle diameter.

**Figure 4:**
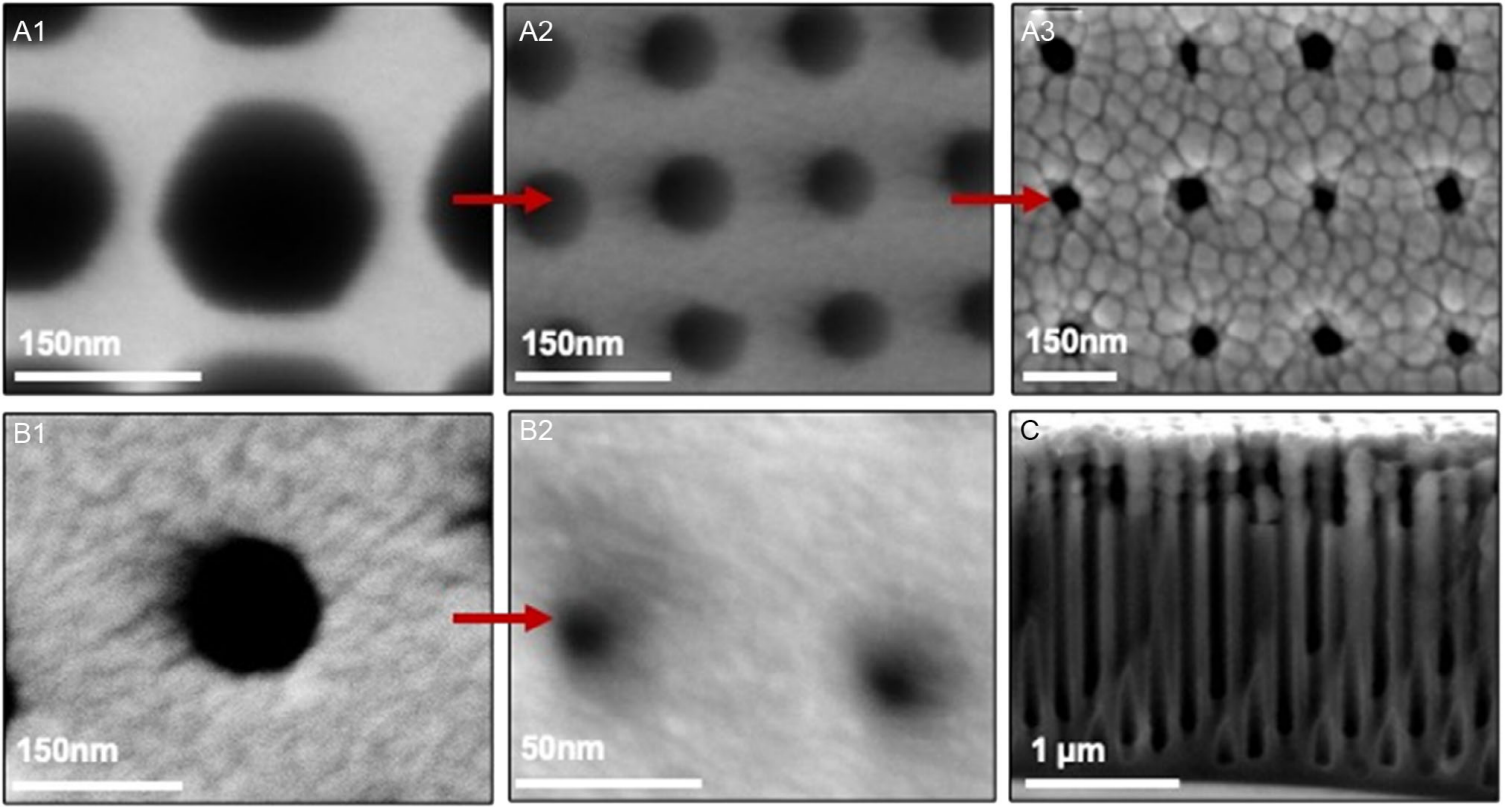
Reducing nozzle size postfabrication (SEM images). A1) Array of 150 nm diameter nozzles defined by EBL and etched by the Bosch process. A2) Nozzles narrowed to 80 nm diameter by depositing 80 nm SiO_2_ (surface thickness) by PECVD. The surface thickness differs from thickness deposited on sidewalls of nozzle due to limited sidewall conformality of PECVD deposition. A3) Nozzles after 20 nm Al_2_O_3_ was deposited by ALD to further reduce nozzle diameter to 30 nm. B1) Array of 150 nm diameter nozzles defined by EBL and etched by the Bosch process. B2) Nozzle array after 60 nm Al_2_O_3_ was deposited by ALD to reduce nozzle diameter to 26 nm. C) Cross‐sectional view of sample A. The SiO_2_ deposited by PECVD is concentrated on the upper ≈500 nm of the nozzles. Full penetration of the Al_2_O_3_ deposited by ALD is evidenced by reduced diameter (110 nm) throughout the nozzle depth. Note that the manual cleaving process caused multiple rows of the nozzle array to be intersected in some areas and uneven features not believed to be inherent to the fabrication process.

To this end, we have developed an additional process for further narrowing the nozzle aperture by performing a PECVD necking and ALD trim (Figure 3g). These processes lead to deposition along the rim of the nozzle, leading to further narrowing. **Figure** **4**a shows an array of test nozzles on a blank wafer which have been reduced from 150 to 80 nm diameter by depositing 80 nm (surface film thickness) SiO_2_ by PECVD (see Supporting Information for recipe), and then further reduced to 30 nm by depositing 20 nm Al_2_O_3_ by ALD (see Supporting Information for recipe). The PECVD‐deposited material is not perfectly conformal to the nozzle sidewalls, so depositing 80 nm film thickness on the surface results in ≈35 nm thickness of material deposited on the nozzle sidewalls.^[^ ^37^, ^38^ ^]^ The SiO_2_ coverage is maximal in the upper part of the nozzle opening and tapers down deeper inside, as shown in Figure 4c. The ALD deposition is highly conformal, so the same amount of material is deposited on the nozzle walls as is deposited on the surface of the substrate. ALD processes are conformal over a wide range of process conditions due to their layer‐by‐layer deposition, thus it is likely that the ALD coverage is uniform throughout opening.^[^ ^37^, ^38^ ^]^ This method has the drawback that the grain size of the film deposited by PECVD is large relative to nozzle diameter and results in nonuniform nozzle diameters and possible clogging but would be appropriate if uniformity is of less concern because necking by ALD deposition alone is a time‐intensive process.

Alternatively, the nozzles may be trimmed solely by ALD. Figure 4b shows an array of test nozzles which have been reduced from 147 nm diameter to 26 nm diameter by depositing 60 nm of Al_2_O_3_ by ALD (see Supporting Information for recipe). We note that while we have demonstrated the fabrication of ≈30 nm nozzles on blank substrates, the smallest nozzle diameter used for depositions using the microevaporators was limited to ≈300 nm. Integrating 30 nm nozzles onto the nozzle head geometry involves additional integration processes which we are currently working through, and which is beyond the scope of the current article.

### 2.3 Microevaporator Loading

To deposit materials, the reservoir of the microevaporator is manually filled with solid material and sealed by mounting the back face of the microevaporator to a 200 μm thick glass cover slide via indium bonding. Indium bonding has been a longstanding practice in areas such as molecular beam epitaxy^[^ ^39^ ^]^ where it has been used to bond substrates to substrate holders due to the low melting point of In (160 °C) and its low outgassing under vacuum. The bond is held by the surface tension of the liquid In between the mating surfaces when the substrates are heated to above the melting temperature of In.

A “black silicon” chip is mounted using low vapor pressure epoxy on the opposite side of the glass. It is used as a heat absorber for radiative heating of the microevaporator and is aligned with the footprint of the microevaporator. Black silicon refers to silicon that has been etched to create a needle‐like roughened surface which results increased light absorption.^[^ ^40^ ^]^ In this work, the black silicon is created by a RIE process (see “Black Silicon” in the Supporting Information). A thermocouple is sandwiched under the black silicon chip to monitor the temperature of the black silicon. We assume that the whole system consisting of black silicon chip and microevaporator is at the same temperature due to the high thermal conductivity of silicon and thin glass, so the thermocouple temperature measurement is taken to be that of the microevaporator.

### 2.4 Scanning Stage Design and Alignment

Figure 1b illustrates the overall scanning stage design, with the microevaporator attached glass slide (blue) mounted on a static stage and the substrate (purple) kept on a three axis nanopositioning stage to be moved during deposition. Depositions are performed under vacuum (≈10^−7 ^kPa) to allow the vapor to travel from the nozzle to the substrate without risk of any collisions with atmospheric gas molecules. A copper cold finger is also attached to the scanning stage to allow for liquid nitrogen substrate cooling during deposition.

Methods of precisely positioning a small, delicate probe within nanometers of a substrate surface have long been practiced in the field of scanning probe microscopy.^[^ ^41^ ^]^ However, the problem becomes more complicated when the atomically sharp tip of the scanning probe is now a flat surface of a finite area, as with the microevaporator. Here, a small relative tilt between the plane of the probe and the plane of the substrate surface limits the minimum distance of the nozzle (centered on the probe plane) to the substrate. To limit this effect, in addition to designing the nozzle geometry described in Section 2.2, the surface of the glass slide is manually aligned to be parallel with the surface of the substrate prior to pump down.

To set the surface of the glass slide parallel to the substrate, we implement a four quadrant segmented photodiode to perform a procedure similar to the initial positioning methods used by Uppuluri et al.^[^ ^42^ ^]^ First, we load the substrate and align the four‐quadrant segmented photodiode sensor to be parallel to the substrate by reflecting an alignment laser from the substrate and centering that signal on the photodiode. Then, we add the glass slide assembly to the system above the substrate such that it is in front of the alignment laser and align the glass slide to the photodiode sensor by tilting the glass slide until the signal is once again centered. The glass slide is coated with gold to allow the laser to reflect back to the sensor and the glass is mounted in the system using a lens mount with three‐point tilt adjustment for tilt (see Experimental Section). This alignment is performed to a measured accuracy of 10^−3^ rad. Thus, with a pillar width of 30 μm, the maximum distance of the nozzle from the substrate is 30 nm when the edge of the pillar is in contact with the substrate.

### 2.5 Temperature Control

To heat the device for deposition, focused light from an LED (see Experimental Section) is used to heat the black silicon while the temperature is monitored by the thermocouple under the black silicon (for placement details, see Section 2.3). As the glass slide is thin (≈200 μm), the black silicon on top of the slide and the microevaporator on the bottom of the slide are assumed to be the same temperature. Due to the low thermal conductivity of glass, heat is not expected to be conducted significantly in the plane of the glass slide, resulting in no conductive thermal interaction between source and the rest of the system when the source is not in contact with the substrate. The substrate is attached to a copper sample holder by vacuum grease (see Experimental Section), and the temperature is regulated either passively using the room temperature copper as a heat sink, or by active cooling by a liquid nitrogen cold finger. The cold finger connects to the copper sample holder by a flexible piece of braided copper.

We use the relationship between *z* position and temperature to calibrate the position of the microevaporator tip relative to the surface of the substrate. The microevaporator is heated by a constant power from the LED while the substrate is raised and lowered to determine the position at which it makes physical contact with the microevaporator. During this touchdown procedure, the distance between hot microevaporator and cold substrate is decreased (using the piezoelectric drive) until a sharp drop in temperature of the microevaporator is observed, which is thought to be caused by the sudden increase in thermal loss due to contact between heated microevaporator and the cold substrate. At sub‐10 μm distances, the prevalence of near‐field radiative heat transfer (NFRHT) over blackbody radiation is known to take place and is enhanced with decreased distance, but those effects are expected to be subtle compared to the effect of contact with the substrate.^[^ ^35^ ^]^ The position at which the sharp drop in temperature is observed is taken to be the position of *z* = 0, where the nozzle head is in contact with the surface of the substrate. The microevaporator to substrate distance is then set by retracting the microevaporator to the position using the piezoelectric drive. The source to substrate distance is verified by analysis of the deposition profiles ex situ, detailed in Section 2.6.3.

### 2.6 Deposition Experiments

#### 2.6.1 Fixed Deposition

**Figure 5:**
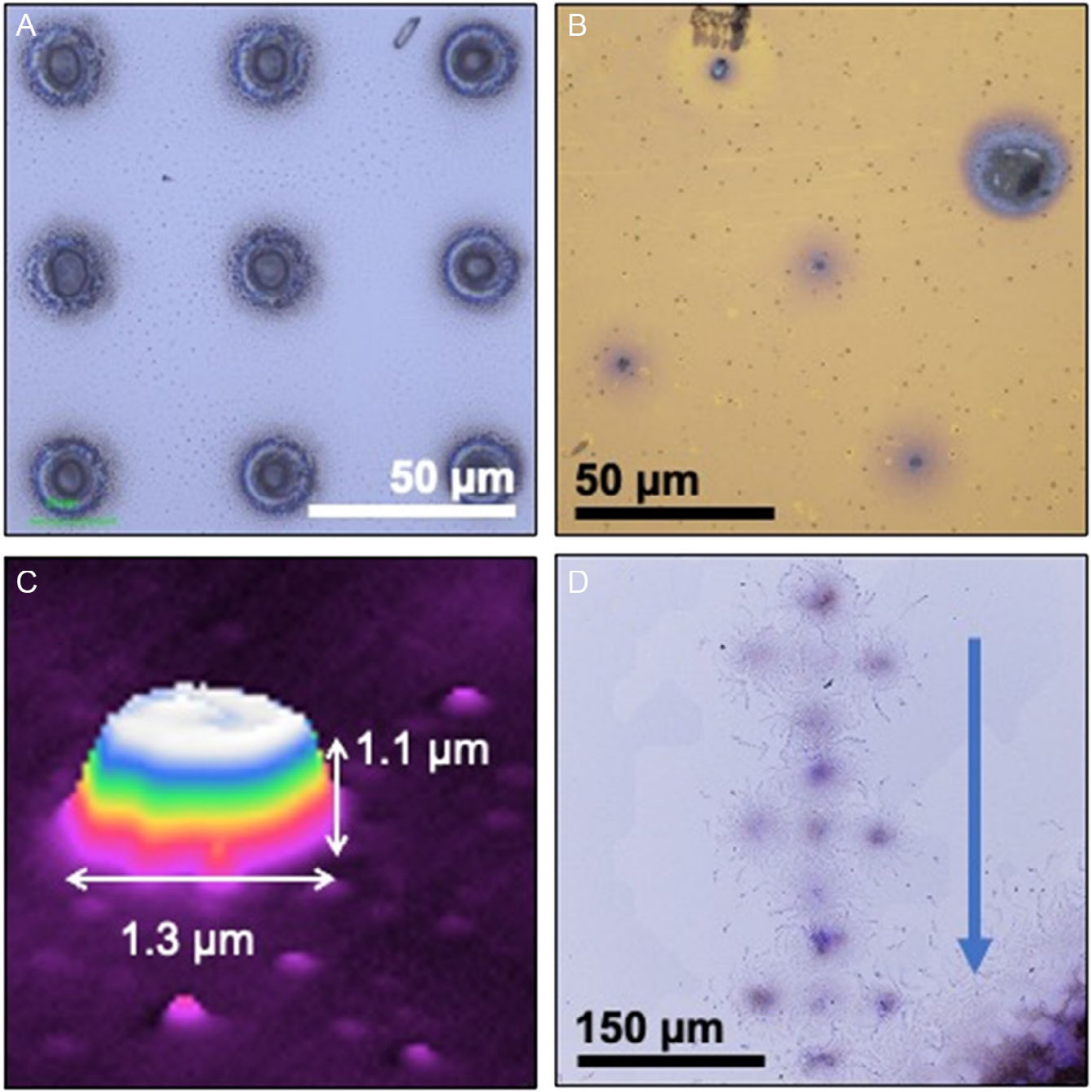
Static depositions of various materials. A) Optical microscopy image of PTCDA deposited on silicon substrate. Deposition performed from a microevaporator with nine 800 nm diameter nozzles on one nozzle head, 150 °C microevaporator temperature, 23 °C substrate, 60 min exposure time. B) Optical microscopy image of zinc deposited on 150 nm Au/7 nm Cr/Si substrate. Deposition performed from device with five nozzle heads each with a single 900 nm diameter nozzle, 290 °C microevaporator temperature, 23 °C substrate, 60 min exposure time. The larger spot on the top right is due to membrane damage on that nozzle head. C) SLCM profile data from Coumarin dot deposited from 900 nm nozzle, 160 °C microevaporator temperature, 23 °C substrate. D) Optical microscopy image of step‐and‐repeat Coumarin deposition, translated in direction indicated by arrow. Deposition performed from five nozzle head device with 900 nm diameter nozzles, 160 °C microevaporator temperature, 23 °C substrate.

The performance of the microevaporators was tested using low vapor pressure evaporants such as coumarin‐6 (“coumarin”), perylenetetracarboxylic dianhydride (PTCDA), and zinc. PTCDA and coumarin were deposited on bare Si <100> substrates, while zinc was deposited on Si <100> with a 7 nm Cr/150 nm Au adhesion layer deposited by electron beam physical vapor deposition. Representative static (i.e., scanning stage held at constant *x*–*y*–*z* position) depositions of PTCDA, zinc, and coumarin are shown in **Figure** **5**. These materials have been vacuum evaporated for thin film deposition by others^[^ ^43^, ^44^, ^45^ ^]^ and have vapor pressures greater than 0.1 Pa at temperatures less than 300 °C. The highest achieved temperature for the microevaporator in our case was 300 °C and was limited by the 300 mW incident optical power of the LED currently used. Higher evaporation temperatures are possible with laser or resistive heating, with the ultimate limit being the decomposition of the silicon‐based microevaporator. Due to this limit, as well as substrate heating caused by increased thermal coupling at high temperatures and close evaporator‐to‐substrate distances due to NFRHT, deposition of low vapor pressure metals is a considerable challenge. However, a large number of metals relevant to microelectronics may be deposited from this device by CVD processes using organometallic precursors whose transport and deposition chemistry do not require high thermal budgets.^[^ ^46^, ^47^ ^]^ A future research aim is to adapt the microevaporator to accept such precursors. The current precursors were chosen for the preliminary proof of concept of the microevaporator due to their low evaporation temperature, air stability, and low toxicity.

In our experiments, the deposited spot size is taken to be an indicator of the source‐to‐substrate distance (see Section 2.6.3). In the deposition shown in Figure 5a, the full width at half maximum (FWHM) measured by scanning laser confocal microscopy (SLCM) of the smallest spot is 10 μm, about 10× the nozzle diameter (0.8 μm), indicating a larger source‐to‐substrate distance compared to the deposition in Figure 5b,c where the FWHM of the smallest spot is 1.5 and 1 μm respectively, less than 2× the nozzle diameter (0.9 μm). The deposition shown in Figure 5c can be viewed as a benchmark, suggesting that depositions from a nozzle in this system can be similar in size to the nozzle from which they are deposited. This leads to the reasoning that shrinking the nozzle diameters farther could result in depositions with nanoscale feature size. The zinc deposition in Figure 5b has one spot which is much larger than the others, which was caused by damage to the top membrane of the nozzle head during handling, resulting in the 10 μm TSV opening depositing directly on the substrate.

A step‐and‐repeat deposition of coumarin is shown in Figure 5d, where the nozzles were stepped along the direction of the arrow in 150 μm steps without recalibrating the source‐to‐substrate distance. The deposited spots become slightly more diffuse and spread out as the microevaporator moves along the surface. This is likely due to angular mismatch between the surface of the substrate and the horizontal translation axis, thus causing a variation in source‐to‐substrate distance between the different depositions. At the upper part of the image, the nozzles are farther away from the substrate, resulting in the slight broadening of the deposition features. We do not believe the differences are due to nozzle clogging because the nozzle is hot, and because we have used the same nozzle repeatedly to deposit over weeks with no evidence of clogging.

We have obtained preliminary statistics on the variation of the deposited spot size from depositions from 0.8 μm diameter nozzles and intended 500 nm source‐to‐substrate distance. Analysis of 25 Coumarin deposition spots from five separate deposition trials from devices containing five nozzles each yielded an average spot size of 1.3 μm with 0.5 μm standard deviation. Variations can arise due to varying nozzle diameter, and source‐to‐substrate distance. We believe that the source‐to‐substrate distance is a key contributor to the variability, and that it may be improved by continuously adjusting the source‐to‐substrate distance during deposition implementing a closed loop feedback control using a sensitive technique such as NFRHT. The radiative thermal losses of the heated microevaporator should vary with distance in the near field because NFRHT, in contrast to blackbody radiation, is a function of the distance between the two objects. The dependence of NFRHT on the distance, *d*, depends on the geometry of the two objects and scales as *1/d* ^2^ between parallel plates such as the nozzle head/substrate system presented in this article.^[^ ^35^, ^48^ ^]^

#### 2.6.2 Writing of Lines

**Figure 6:**
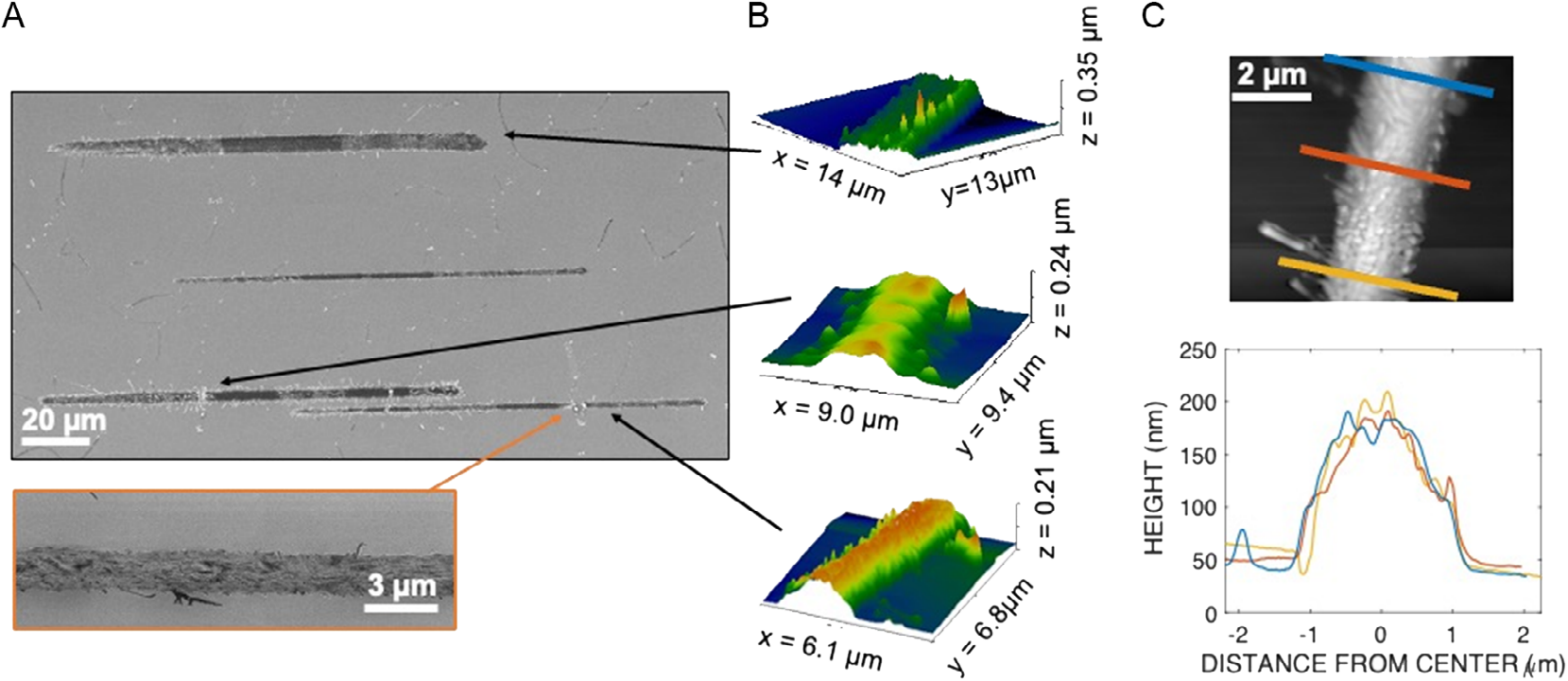
A) SEM images of Coumarin deposited from 160 °C microevaporator with 700 nm to 300 nm diameter nozzles and <1 μm source to substrate distance, 23 °C substrate temperature, 5 nm s^−1^ translation speed. Resulting linewidth 8 μm to 2 μm and thickness 200–300 nm. B) Line profile data collected by atomic force microscopy (AFM). The images correspond to lines noted by the black arrows. C) AFM height profiles at three points 2 μm apart along one of the lines shows uniformity in the profile along this span of the line.

Lines were written with coumarin, evaporated at 160 °C (**Figure** **6**a). In Figure 6a, initial contact with the substrate surface was determined by thermal touchdown, as described in Section 2.5, and then the source‐to‐substrate distance was increased by 500 nm for deposition. Lines were written with a translation speed of 5 nm s^−1^. Variation in linewidths between the different lines in Figure 6a occurs due to variation in nozzle diameters that arises from the PECVD/ALD nozzle trimming process as described in Section 2.2.1. The linewidths were between 2 and 8 μm; the film thicknesses of the lines ranged from ≈200 to 300 nm.

In contrast to the large cross‐sectional variation between lines from different nozzles (Figure 6b), the cross‐sectional profiles are fairly uniform along the length of each individual line, as observed visually in Figure 6a and in cross‐sectional view over a representative line segment in Figure 6c. Some variations in profile along the lines appear to be caused by acicular grain patterns of the coumarin crystals within the line, which can be seen on the inset in Figure 6a. Selective exposure to the SEM beam during higher magnification imaging of some sections of the lines (e.g., Figure 6a, orange outlined image) caused such sections to appear darker in the image due to charge accumulation in the nonconductive material and is an artifact.^[^ ^49^ ^]^ The lines were written in the right to left direction, and the taper on the left side is due, in part, to angular mismatch between the substrate surface and translation axis. We do not believe this is due to nozzle clogging (this is further discussed in Section 2.6.1 regarding the deposition shown in Figure 5d).

The dependence of deposition width on source to substrate distance can be used to check that the source‐to‐substrate distance was below a certain threshold and/or held constant during writing lines. These results are discussed in Section 2.6.3, in the context of comparison to calculated and simulated results.

#### 2.6.3 Deposition Profiles Analysis

When depositing from an evaporative surface point source, the deposited mass per unit area is described as (Knudsen's cosine distribution law).^[^ ^50^ ^]^

(1) $$\frac{d{\bar{M}}_{s}}{dA_{s}}=\frac{{\bar{M}}_{e}\text{cos}\phi \text{cos}\theta }{2\pi r^{2}}\;$$

${\bar{M}}_{e}$ is the total evaporated mass, *r* is the line from the source to the substrate, *ϕ* is the angle between *r* and the source normal, and *θ* is the angle between *r* and the substrate normal (see Supporting Information, Figure S4 for illustration). Equation (1) is formulated for the case where the source‐to‐substrate distance is much larger than the source size, as it is in a traditional deposition system with an evaporative source.

**Figure 7:**
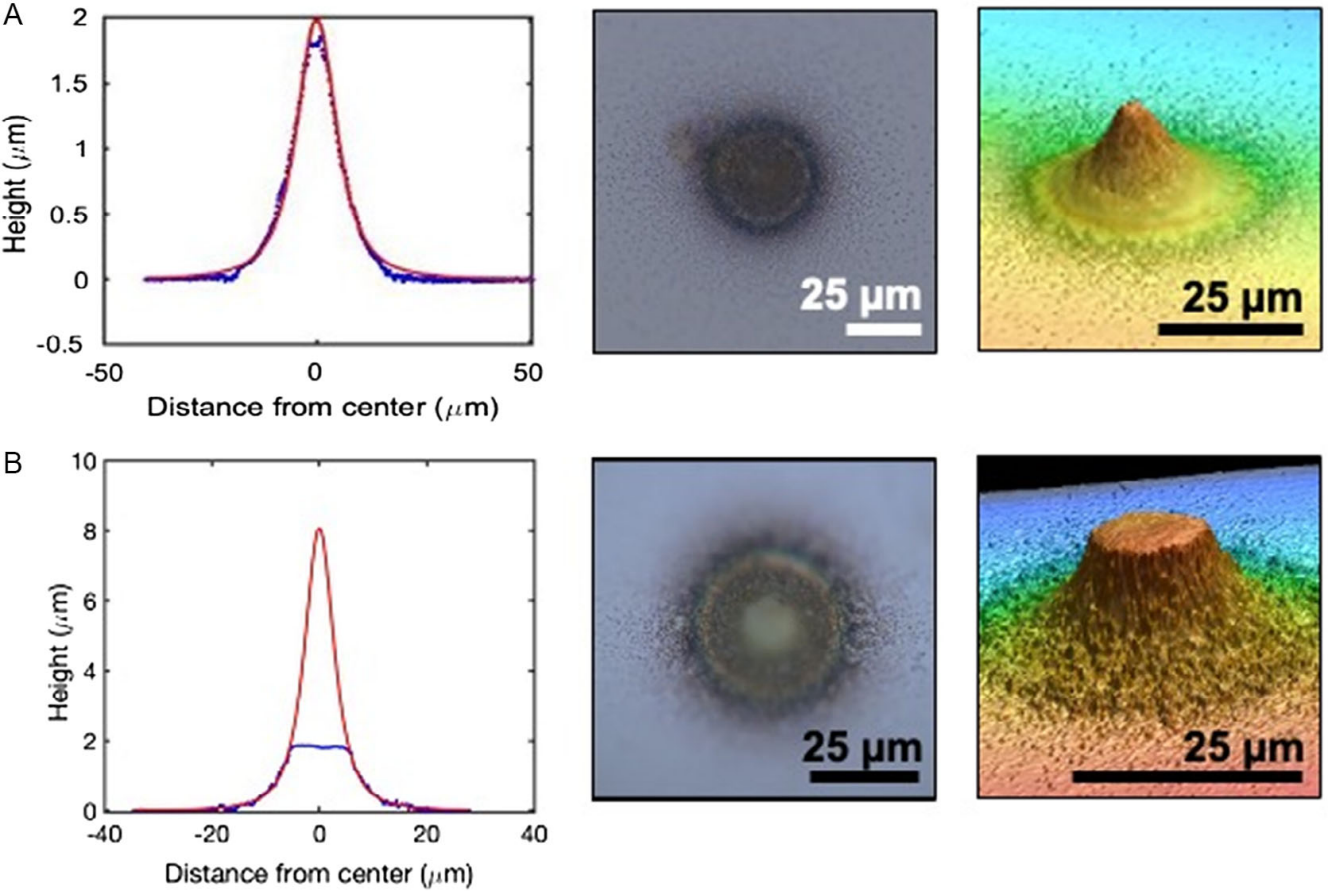
Deposition profiles. A) Fitted source‐to‐substrate distance, *z* = 7.2 μm. As this deposition was performed at a relatively far distance, the fitted curve corresponds well with the measured profile. B) Fitted source‐to‐substrate distance, *z* = 4.2 μm. The flat shape cannot be fit well with Equation (1). Left: Profiles from *r* = 1000 nm devices measured by SLCM (blue) with fitted line (red) according to the cosine law (Equation (1)). (center) Corresponding optical microscopy image of deposition. Right: Corresponding SLCM 3D image, with aspect ratio in out‐of‐plane direction enhanced for viewing sidewall shape.

In **Figure** **7**a, an example of a deposition profile (blue) that fits Equation (1) well, along with a fitting (red) with *n* = 0 and *z* = 7.2 μm to Equation (1), is shown. However, many depositions were also observed that had flattened tops, as shown in Figure 7b, which did not result in good fitting to Equation (1). The deposition in Figure 7a was performed at a distance large enough such that Equation (1) describes the flux well, but the deposition in Figure 7b was performed at a smaller distance, such that the assumptions held in Equation (1) no longer apply.

**Figure 8:**
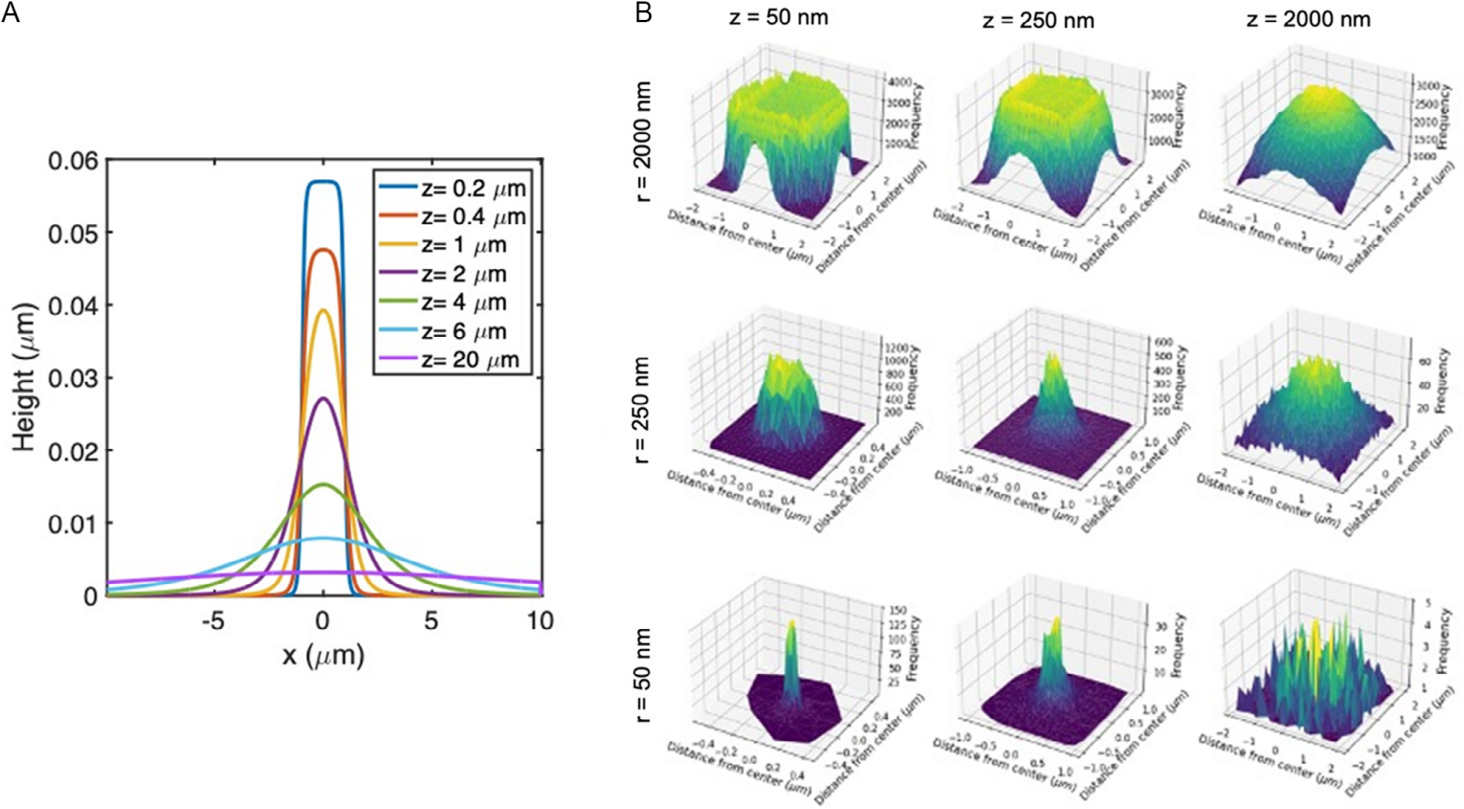
A) Height profiles for *r* = 1000 nm plotted from calculation by adaptation of cosine distribution law. B) Height profiles plotted from DSMC simulations for nozzle radius *r* = 50, 250, and 2000 nm and source‐to‐substrate distance *z* = 50, 250, and 2000 nm.

At closer distances, two major differences arise between our system and the standard point source approximation: 1) the source size (nozzle diameter), and 2) the height of the accumulated deposition become comparable to the source‐to‐substrate distance, *z*. We account for (1) by adding contributions (using Equation (1)) as if there is a collection of surface sources evenly distributed over the width of the nozzle. Each point along the nozzle is assumed to emit a flux pattern following Equation (1). We address (2) by accumulating the deposition on the surface layer by layer and accounting for shadowing effects caused by line‐of‐sight considerations. Calculations which augment Equation (1) to include compensation for (1) and (2) are performed numerically in MATLAB using a 2D model (see Supporting Information for details). The resultant profiles from a 2 μm diameter nozzle are shown in **Figure** **8**a. A flat top shape begins to occur as we lower to *z* positions z<<r, as can be seen by the *z* = 0.2 μm line in Figure 8a. The deposition profile becomes flat on the top as the nozzle approaches contact with the substrate, an extreme which would result in a perfectly flat deposition inside the nozzle with vertical sidewalls and no deposition outside the nozzle area.

The evolution of the deposition profile with source‐to‐substrate distance during deposition is further studied using DSMC simulations performed using Stochastic Parallel Rarefied‐gas Time‐accurate Analyzer (see Supporting Information).^[^ ^51^ ^]^ In this model, the molecules are treated as billiard balls between collisions with the resultant trajectories following gas–gas or gas–surface collisions determined stochastically. This simulation allows calculation of the trajectory of molecules as they travel through and exit the inner geometry shown in Figure 1a, experiencing collisions with surfaces and other molecules in the reservoir, TSV, and nozzle. The molecules are calculated to experience diffuse reflection on surfaces (as opposed to specular reflection), so that the molecules that collide with solid surfaces are adsorbed and then reemitted with a new angular velocity distributed according to its thermal energy after interacting with the surface.^[^ ^51^ ^]^

A 3D model was developed (see Supporting Information, Figure S3 for illustration) based on the dimensions outlined in Figure 1a, except the depth of the reservoir was shortened to reduce the overall size of the simulation. In this simulation, the reservoir is populated with gas molecules (monatomic, with weight and Bohr radius provided as inputs) at 500 K, 10^−2^ kPa, and the areas outside the reservoir are set to be void of molecules. When the simulation is run, it is first allowed to equilibrate to a steady state of molecular flux exiting the nozzle. Following equilibration, the molecular flux is counted on a surface set at a distance *z* away from the nozzle opening with radius *r* over the span of 1 ms.

The plots in Figure 8b show the concentration of molecular flux across the substrate surface for various values of *r* and *z*. For these plots, we used the atomic weight and Bohr radius corresponding to Zn. Simulations were also run with the molecular weight and estimated molecular diameter of Coumarin, resulting in similarly shaped depositions with a different rate of accumulation due to the larger size of the molecules. The DSMC results concur that reducing nozzle size and improving control over the source‐to‐substrate distance will lead to more localized depositions and thus improved linewidths (Figure 8b). Regarding the qualitative shape of the deposition, DSMC simulations agree with the earlier discussed calculations based on the cosine law equation, showing that the deposition profiles begin flattening at the top as the source‐to‐substrate distance is reduced (Figure 8b).

**Figure 9:**
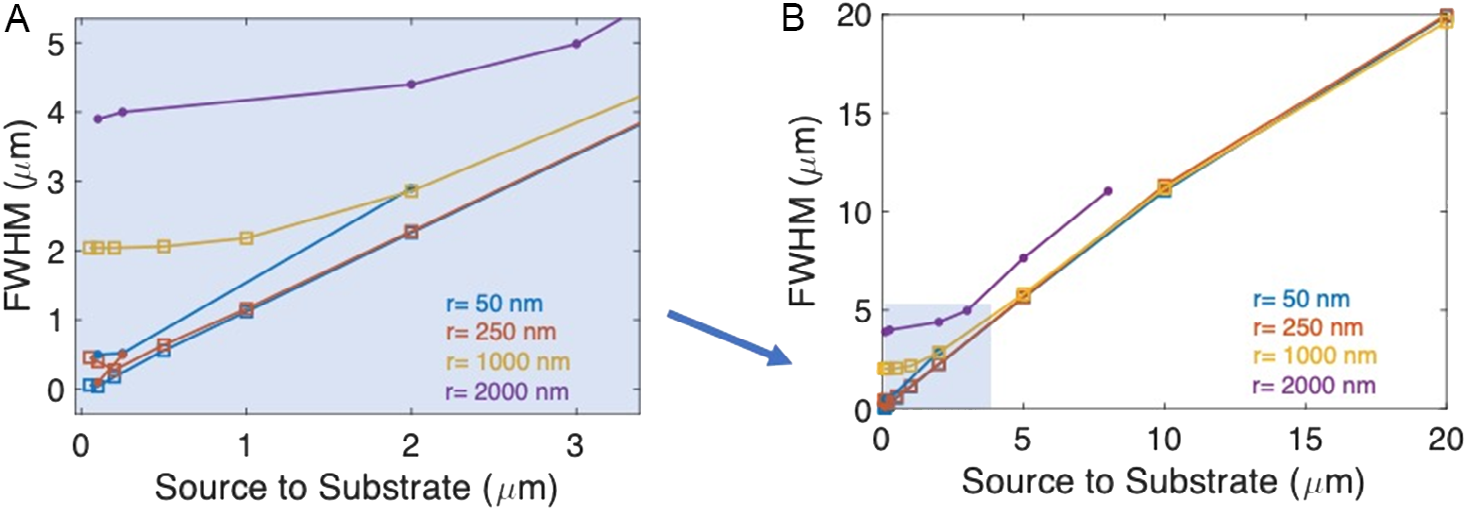
Summary of FWHM versus source‐to‐substrate distance for various nozzle sizes. The colors of the lines indicate the nozzle radius (see legend). DSMC‐simulated points are denoted by closed circles; cosine distribution law‐derived points are denoted by open squares. A) Zoomed in plot for source‐to‐substrate distances up to 3 μm. B) Full plot showing up to 20 μm source‐to‐substrate distance.

The resulting FWHM of depositions with varying nozzle size and source‐to‐substrate distance as simulated by DSMC are plotted in **Figure** **9** (closed circles) alongside the results from the previously discussed cosine distribution law analysis (open squares). At large distances, the deposition system approaches the scale condition which is modeled by Equation (1), thus the FWHM versus source‐to‐substrate distance relationship becomes linear. At lower source‐to‐substrate distances, the plotted curve flattens as the FWHM approaches an absolute minimum (the deposition width cannot be smaller than the nozzle size). Note that, in the FWHM versus source distance graph (Figure 9), the FWHM appears to increase at very small source‐to‐substrate distances. This is an artifact of the evolving profile shape of the deposition from rounded to increasingly flat‐topped.

In summary, the DSMC simulations and cosine distribution law‐based results agree on a source distance versus FWHM trend which flattens out when *z < r* and increases linearly for *z » r*. The smallest possible spot size will be comparable to the nozzle diameter.

When comparing the results between the cosine distribution law adaptation and DSMC simulations in Figure 9, there is a slight difference in results, especially at smaller values of *z*, where the DSMC values tend to be a few hundred nm larger than the cosine distribution law results. This is likely due to the cosine distribution law ignoring the effects of gas–surface collisions at the edge of the nozzle on the exit trajectories of molecules, while the DSMC method does account for this effect.

Both methods assumed that surface diffusion of the deposited adatoms on the substrate surface is negligible. As a result, the simulations should be regarded as a “best case” resolution, one where each adatom sticks to the substrate where it lands. In a realistic system, adatoms are expected to diffuse across the surface depending on temperature and atomistic interactions with the surface which is highly substrate and evaporant dependent.^[^ ^52^, ^53^, ^54^ ^]^ Thus, when comparing to experimental results this modeling can be used as an estimate of the upper bound of the source‐to‐substrate distance, but not as an absolute description of the deposition profile.

Depositions with both rounded and flattened tops were observed in experiment, with flattened tops appearing for smaller source‐to‐substrate distances. In both the cosine distribution law‐based and DSMC‐simulated solutions, the edges of the flat top correspond directly with the size of the nozzle. However, in experiment, the edges of the flat tops are often much further from the center of the deposition than in the simulated results and the flat top dimension can be several times the width of the nozzle. For example, the deposition with a 10 μm wide flat top from a 2 μm wide nozzle is shown in Figure 7b. On the other hand, in some depositions, such as that shown in Figure 5c, the width (1 μm) does correspond closely with the nozzle size (0.9 μm). The reason for this is not understood. Neither of the models accounts for adatom migration on the substrate surface—one possibility might be alteration of the deposited profile cause by adatom surface diffusion‐driven redistribution. This may be exacerbated by localized heating of the substrate due to NFRHT. Observation of acicular crystalline structures within some deposition spots indicates adatom mobility following deposition, which supports this hypothesis. A second possibility may be “creep” of the evaporant radially outward from nozzle and along the nozzle head surface leading to an effectively larger diameter source.

Regardless, we can use the measured FWHM of depositions and compare them to simulations to determine an upper bound for the source‐to‐substrate distance because these effects will only lead to a larger FHWM (therefore larger estimated source‐to‐substrate distance) than predicted. For example, the dot shown in Figure 5c was deposited by a *r* = 450 nm nozzle, resulting in a FWHM of 1.3 μm. Matching this result to the graph in Figure 9, we obtain a maximum source‐to‐substrate distance of 1 μm because a *r* = 250 nm nozzle at 1 μm will produce a FWHM > 1 μm and a *r* = 1000 nm diameter nozzle deposition has FWHM > 2 μm at *z* = 1 μm. We can also apply the knowledge in Figure 9 to a line deposition. The line shown in Figure 6c has a FWHM of 2 μm and is from a nozzle with *r* = 150 nm. This nozzle falls between the *r* = 250 nm and *r* = 50 nm curves in Figure 9, both of which have *z* < 2 μm for 2 μm FWHM. Therefore, we reason that the deposition in Figure 6c was deposited from a nozzle held a distance less than 2 μm away from the substrate. Furthermore, as this is in the linear part of the curve for both *r* = 50 nm and *r* = 250 nm, we know that the linewidth should vary directly with any variation in source‐to‐substrate distance during write. If the line's width is constant over its length, this means that the write operation was performed at a constant source‐to‐substrate distance along its length.

## 3 Conclusion

We have described the fabrication and use of a novel silicon‐based microevaporator developed for direct‐write technology applications. We have successfully demonstrated a process flow for the fabrication of the microevaporator with nozzles sizes down to 300 nm, as well as shown a route to fabricating nozzles as small as 30 nm by fabricating 30 nm nozzles on a blank wafer. We have then integrated the microevaporator with remote heating by LED, a piezo‐driven near‐field scanning stage in vacuum, and developed a novel way to set source‐to‐substrate distance by thermal interactions for deposition onto a substrate via a direct‐write approach. The capabilities of the microevaporator were demonstrated by initial experiments with depositions of low‐temperature PVD materials, demonstrating the deposition of spots and lines with 1–10 μm widths from microevaporator nozzles in the 300 nm to 2 μm range. Finally, using two approaches—one based upon Knudsen's cosine law and another based upon a Monte Carlo approach—we have developed simulations for predicting the direct‐write deposition profiles and resolutions and compared them to the experimental results.

The simplicity of an all‐in‐one device which contains both evaporation reservoir and nozzles would allow quick integration into any preexisting vacuum system that allows for a scanning probe and heat source. As this microevaporator is fabricated from silicon, the vast library of silicon‐based fabrication technologies is available for modification and development of the device, including narrowing of the nozzle diameter. The deposited feature size demonstrated in this article is already appropriate for applications in HI (two orders of magnitude smaller than the current state of the art for printed interconnects on organic circuit boards); there only needs to be added a capability to deposit the required materials for this application. Additionally, from these benchmark results, we believe that much smaller feature sizes will be possible with this system for applications in 3D direct‐write chip manufacturing.

Future challenges include demonstrating scalability for large systems, deposition compatibility with high elemental vapor pressure materials, and better deposition feature size control. Scalability can be addressed via the fabrication of massively parallel arrays of evaporators—similar to the approach of Millipede technology^[^ ^55^ ^]^ that was developed for indentation writing of magnetic bits for memory. Extending our approach to connect the microevaporator reservoir to feed‐ins for gas‐phase precursors for CVD and ALD will simplify flow control and increase versatility by opening up the technique to a range of materials for which robust precursor chemistries for deposition exist. Source‐to‐substrate distance has been identified as an important factor in the effort to create more predictable, uniform depositions, therefore developing noncontact methods by which to determine and maintain source‐to‐substrate distance is an important next step to improve this technology. Precise understanding and measurement of temperature changes corresponding to source‐to‐substrate distance due to NFRHT is one possible approach. Our current work is aligned along the directions of adapting to CVD deposition chemistries and NFRHT.

## 4 Experimental Section

4.1

4.1.1

##### Cleanroom Processing

Electron beam lithography was performed with JEOL 8100FS and optical lithography was performed with Heidelberg MLA150 Maskless Lithography. RIE etches were performed with RIE Oxford PlasmaLab 100. Black silicon was produced by etching with Plasma‐Therm Versaline Deep Si RIE. PECVD deposition was performed with Oxford Plasmalab 100 inductively coupled PECVD. ALD was performed with Arradiance Gemstar.

##### Deposition Experiments

Ted Pella Micro Cover Glass 22 × 22 mm, No.1 slides were coated in 7 nm Cr and 150 nm Au by Angstron EvoVac Electron Beam Evaporator. On the opposite side, an approx. 4 mm × 4 mm black silicon chip (fabricated by RIE, see Experimental Section: Black Silicon) and NiCr–Ni thermocouple (Goodfellow) were adhered with Loctite AE1C two part epoxy which was cured on a hotplate at 121 °C for 30 min. Loctite AE1C was chosen because it has low outgassing properties at temperatures up to 300 °C. The microevaporator was affixed to the gold coated side of the glass slides directly opposite the black silicon chip by indium (Indium Co.) on a hotplate at 180 °C. Evaporation materials were purchased from Sigma–Aldrich and used as received. The alignment system consists of a ThorLabs PL201 laser, ThorLabs PDQ80A Quadrant Detector Sensor Head, 50/50 mirror, and optomechanical parts purchased from ThorLabs. Nanopositioners ECS50 × 50/NUM/UHV were purchased from Attocube and attached to a custom‐designed stainless steel stage which was fabricated by the University of Chicago Central Shop. A mounted LED (ThorLabs M660L4 with controller ThorLabs DC2200) was used for heating the microevaporators. All deposition trials were performed under vacuum in the range of 10^−5^–10^−7 ^kPa. Custom cold finger purchased from McCallister Technical Services. Vacuum pumps used were: Kurt Lesker KJLC‐RV Rotary pump, Pfeiffer TPH420 Turbo Pump, Agilent Vaclon Plus 75 Ion Pump, Vacuum pressure measured with Kurt Lesker 275 series Convection Vacuum Gauge and 392 Series Ionization Vacuum Gauge. Keithley DMM500 was used to read thermocouple for thermal measurements. Vacuum electrical feedthroughs were purchased from AccuGlass.

##### Characterization

To view the etch cross section, the microevaporators were FIB milled and imaged by FEI Nova 600 NanoLab. The depositions were imaged by either FEI Nova 600 NanoLab or Carl Zeiss Merlin SEM, and 3D laser confocal measurements were performed using Olympus OLS5000 LEXT. Height measurements of the depositions were also performed with a Bruker Multimode 5 atomic force microscope.

## Conflict of Interest

The authors declare no conflict of interest.

## Supporting information

Supplementary Material

## Data Availability

The data that support the findings of this study are available from the corresponding author upon reasonable request.
