# Supplementary material for: Nanofabrication and Demonstration of a Direct‐Write Microevaporator
Source: Small Sci. 2023 Dec 22;4(2):2300121. doi: 10.1002/smsc.202300121 (PMC11935200; doi:10.1002/smsc.202300121)
Supplement: Supplementary file 1 — Supplementary Material [file SMSC-4-2300121-s001.pdf]

SUPPORTING INFORMATION for Nanofabrication and Demonstration of a Direct-Write Microevaporator

<u>Section S1: Microevaporator Fabrication Step-by-Step</u>

In the following, we refer to the device layer side as the front side, and the reverse side as the back side.

The nozzle openings and wafer alignment marks are first defined on the front side by EBL. They are then etched by a Bosch etch (see S.I., Bosch 1) which terminates on the SiO_2 layer (**Figure 3a**). The SiO_2 layer serves as an etch stop since the Bosch etch is highly selective (up to >100:1) in etching Si over SiO_2[38]. This Bosch etch is optimized to leave a smooth scalloped surface (undulation less than 50 nm) on the etched walls. This preserves the as-patterned nozzle dimensions. This method allows for the creation nozzle diameters down to 100 nm in diameter and with a depth that is equal to the top silicon layer thickness of the SOI wafer.

Next, the TSVs are patterned in 12 μm thick AZ4620 by optical lithography on the backside and are aligned with the nozzle openings by backside alignment to the alignment marks which were defined during the first step. The 10 μm diameter TSVs are etched by a Bosch etch (see S.I., Bosch 2) to a depth of 200 μm (**Figure 3b**). This etch does not touch down to the oxide because these openings will also be exposed during the subsequent reservoir etch. The depth of the TSV etch is optimized such that the TSVs will touch down on the SiO_2 layer during the subsequent etch that defines the 150 μm deep reservoir.

To form the reservoir, 8 μm thick AZ4620 resist is patterned by optical lithography with 150 μm by 150 μm squares on the backside overlapping the TSVs and Bosch etched (see S.I., Bosch 2) down 150 μm. The TSV etch continues during this step, resulting in touchdown on the oxide

layer. Cleaving lines between individual dies are also patterned and etched during this step (**Figure 3c**). Once the TSVs have landed on oxide, the oxide is also etched from the backside by RIE (see S.I., Oxide Etch) to open the nozzle to the TSV (**Figure 3d**).

The shaping of the nozzle head structure is performed at the end to reduce risk of breakage during fabrication. The 30 μm diameter circular head is patterned on the frontside of the wafer in 8 μm thick AZ4620 by optical lithography. This pattern is prone to lift off during development since there are a lot of edges relative to overall surface area, so poly(methyl methacrylate) (PMMA) is used underneath the AZ4620 to promote adhesion. A Bosch etch (see S.I., Bosch 3) is performed to etch through the 2 μm thick top silicon, followed by RIE to etch through the 2 μm SiO_2 layer (see S.I., Oxide Etch). Lastly, a Bosch etch (see S.I., Bosch 3) is used to etch the remaining 47 μm, resulting in a 50 μm tall pillar on top of which the nozzle is situated (**Figure 3e**).

At this point in the process, all etches are complete and the membranes holding individual dies together on the wafer are only 75 μm thick along the cleaving lines and 175 μm thick elsewhere, so the dies separate when manual pressure is applied (**Figure 3f**).

Section S2: Fabrication challenges

The complex geometry of the microevaporator pushes the limits of nanofabrication. The nozzle must be positioned on a raised nozzle head structure to minimize thermal interaction between the body of the microevaporator and the substrate. Since the pillar is 50 μm tall, the body of the microevaporator is held at a significantly large distance from the substrate relative to the distance at which near field thermal interactions begin to take place (~5 μm for 300 ºC).[41] The fabrication of this high aspect ratio annular nozzle head is challenging in three aspects: etching high aspect ratio features, avoiding exfoliation of the 2 μm top silicon layer which contains the nozzle during this procedure and aligning layers from all four lithography steps.

The TSV has an aspect ratio between 1:30 and 1:13 depending on the depth of the reservoir and the nozzles have an aspect ratio up to 1:13 for the 150 nm diameter nozzles. For features with extreme aspect ratios, the Bosch recipe must be tuned since the etch rate decreases throughout the etch due to the difficulty of entering and exiting of the reactants and by-products respectively in and from the high aspect ratio feature.[42] **Figure SI1a** shows a cleaved cross section of a sample array of 10 μm TSVs etched through 400 μm thick silicon to tune the recipe for the TSV etch in step B (Figure 3b). **Figure SI1b** shows a sample array of 200 nm TSVs etched through 2 μm silicon, as is done to form the nozzle in step A (Figure 3a). Since the Bosch process has excellent selectivity against SiO_2, some overetch is allowed. However, too much overetch results in “footing”, or spreading of the etched feature at the bottom, which reaches the outer wall of the nozzle head, resulting in membrane loss as seen in **Figure SI1c**.

In addition to tuning for high aspect ratio vias, the Bosch process was also tuned for the geometry of the pillars such that they have vertical sidewalls. **Figure SI1d** shows the result when

the same recipe used to etch the TSV is used to etch the nozzle head pillars. The resultant undercut reached the TSV inside the nozzle head and caused the structure to collapse. The nozzle Bosch etch also differs from the TSV etch in that it is tuned to result in extremely small scalloping (shown in Figure 1a) since the ~1 µm scallop size of the TSV and pillar etches are larger than the intended feature size of the nozzles.

The wall between the TSV and the outside of the pillar is 10 µm thick, and therefore was expected to survive a wet etch in buffered oxide etch (BOE) for the time required to etch the 2 µm total vertical oxide etch in steps D and E of the process flow (Figure 3). BOE is often preferable to RIE as it is faster and requires less expensive equipment. In practice, using BOE for this etch resulted in exfoliation of the top silicon containing the nozzle (**Figure SI1e**). We found that the capillary force in the crevice between the top silicon membrane and the oxide layer can cause a lateral etch of more than 15 µm during the time needed to etch 2 µm vertically (**Figure SI1f).** Thus, the use of RIE for the oxide etch steps was necessary to avoid this issue.

The EBL-defined nozzles must be the first step of the process so that all subsequent steps will be aligned to the layer that contains the nozzles, which means that the alignment marks are defined by a 2 µm-deep etch in the top silicon during step A in Figure 3. The resulting alignment marks are challenging to use due to the accumulation of resist in the trenches and poor contrast during alignment, which can result in misalignment of some features by several microns. Figure SI1c-e show misalignment due to this challenge, causing the TSV to be off center in the nozzle head structure.

Figure SI1. Examples of fabrication challenges that need careful adjustment in the fabrication process flow: images acquired by SEM. A) Cleaved cross sectional view of array of 10 µm vias

in 400 μm silicon demonstrating the high aspect ratio etch achieved to form TSV. B) Cleaved cross sectional view of array of 200 nm vias in 2 μm silicon to demonstrate the nozzle etch recipe results, which require a different recipe than the TSV etch shown in A, such that the scalloping in the nozzle is minimized to maintain sub-100 nm dimensions. C) Nozzle head with top silicon released due to excessive over etch of the TSV from the backside. D) Nozzle head array resulting from improperly tuned etch causing undercut reaching the TSV, leading to failure of the structure. Additionally, misalignment of TSVs relative to nozzle head structure can be seen. E) Nozzle head with loss of top silicon due to BOE over etch. Some remaining oxide can be seen as a rough, raised layer which begins ~3 μm from the outer edge of the nozzle head. The TSV is off center due to lithography misalignment. F) Severe undercut of oxide layer caused by BOE capillary forces. The top silicon is suspended above a gap caused by the >15 μm deep undercut of the SiO_2 layer during BOE wet etch.

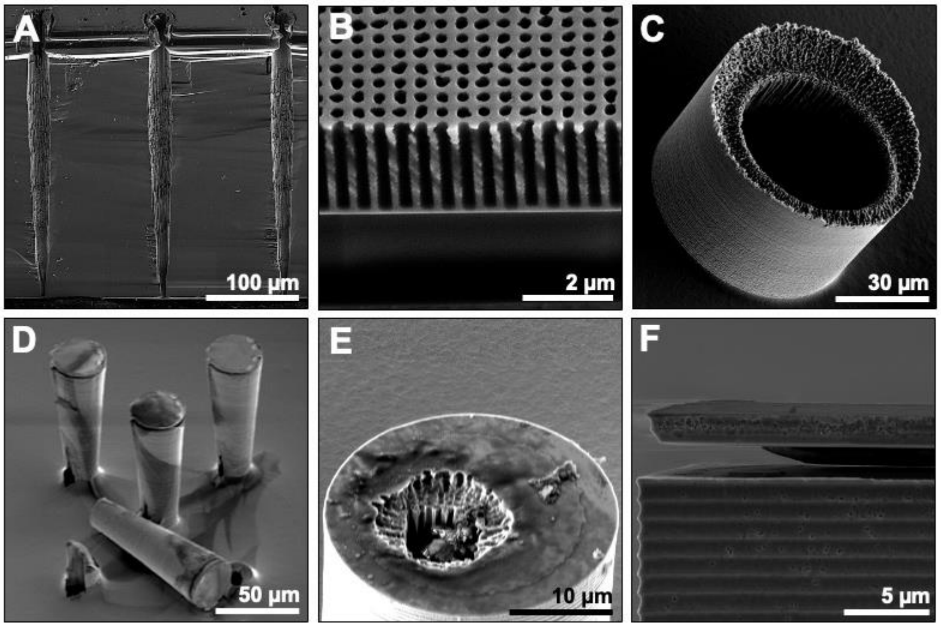

Section S3: Etch Recipes

Bosch 1

Cryo T = 0 C

Pressure = 30 mTorr

time	HF	ICP	SF6	C4F8	Note
3 s	10 W	700 W	1 sccm	100 sccm	Deposit
5 s	30 W	700 W	10 sccm	80 sccm	Etch
3 s	10 W	700 W	1 sccm	100 sccm	Deposit
5 s	30 W	700 W	20 sccm	70 sccm	Etch
3 s	10 W	700 W	1 sccm	100 sccm	Deposit
5 s	30 W	700 W	30 sccm	60 sccm	Etch
3 s	10 W	700 W	1 sccm	100 sccm	Deposit
5 s	30 W	700 W	40 sccm	50 sccm	Etch
3 s	10 W	700 W	1 sccm	100 sccm	Deposit
5 s	30 W	700 W	50 sccm	40 sccm	Etch
3 s	10 W	700 W	1 sccm	100 sccm	Deposit
5 s	30 W	700 W	60 sccm	30 sccm	Etch
3 s	10 W	700 W	1 sccm	100 sccm	Deposit
5 s	30 W	700 W	70 sccm	20 sccm	Etch
3 s	10 W	700 W	1 sccm	100 sccm	Deposit (repeat)
5 s	30 W	700 W	80 sccm	1 sccm	Etch (repeat)

Bosch 2

Cryo T = 15 C

Pressure = 30 mTorr

time	HF	ICP	SF6	C4F8	Note
5 s	10 W	700 W	1 sccm	100 sccm	Deposit (repeat)
7 s	30 W	700 W	80 sccm	1 sccm	Etch (repeat)

Bosch 3

Cryo T = 15 C

Pressure = 30 mTorrr

time	HF	ICP	SF6	C4F8	Note
5 s	10 W	500 W	1 sccm	100 sccm	Deposit (repeat)
7 s	30 W	500 W	80 sccm	1 sccm	Etch (repeat)

Oxide etch

Cryo T = 20 C

Pressure = 10 mTorr

time	HF	ICP	O2	CHF3	Note
	100 W	0 W	2 sccm	50 sccm	13.3nm/ min etch

Black silicon

Spool T = 170C, Lid T = 140C, Liner T = 70C, Heat Exchanger T = 15C

Pressure = 10 mTorr

time	RF	ICP	Ar	O2	C4F8	SF6	Note
10 s	0	0	30 sccm	0	75 sccm	150 sccm	
5 s	1000V, Waveform 1	1500 W	30 sccm	0	75 sccm	150 sccm	
5 min	50V, Waveform 1	1200 W	0	50 sccm	0	70 sccm	

PECVD SiO_2

Heater Table T = 100 C, Chiller T = 5 C

	RF	ICP	N_2O	SiH_4	Note
	0	1200 W	20 sccm	8.5 sccm	Growth Step

~8 nm SiO_2 film growth (on flat surface) per minute

ALD Al_2O_3

Platen T = 175 C, Chamber Heat T = 175 C, Manifold 1 T = 115 C, Manifold 2 T = 120 C

Step	Label	Device	Action	Value	Branch
**1**		**Pump**		**0.000000**	
**2**		**Expo_Heater**	**Set_to_On**	**0.000000**	
**3**		**MFC_Flow**	**Set_to_Value**	**200.000000**	
**4**		**Manifold_1_Heat**	**Set_to_Value**	**115.000000**	
**5**		**Manifold_2_Heat**	**Set_to_Value**	**120.000000**	
**6**		**Platen_Heat**	**Set_to_Value**	**175.000000**	
**7**		**Chamber_Heat**	**Set_to_Value**	**175.000000**	
**8**		**Chamber_Heat**	**Wait_Until_Set_Point_+/-**	**3.000000**	
**9**		**Delay_(Sec.)**		**300.000000**	
**10**	**LoopStrt**	**MFC_Flow**	**Set_to_Value**	**40.000000**	
**11**		**MFC_Flow**	**Wait_Until_<**	**50.000000**	
**12**		**EXPO_Actuator**	**Set_to_Closed**	**0.000000**	
**13**		**Delay_(Sec.)**		**0.200000**	
**14**	**TMA**	**ALD_2_Actuator**	**Pulse_(mSec.)**	**22.000000**	
**15**		**Delay_(Sec.)**		**0.200000**	
**16**		**EXPO_Actuator**	**Set_to_Open**	**0.000000**	
**17**		**MFC_Flow**	**Set_to_Value**	**100.000000**	
**18**		**Delay_(Sec.)**		**28.000000**	
**19**		**MFC_Flow**	**Set_to_Value**	**40.000000**	
**20**		**MFC_Flow**	**Wait_Until_<**	**50.000000**	
**21**		**EXPO_Actuator**	**Set_to_Closed**	**0.000000**	

**22**		**Delay_(Sec.)**		**0.200000**	
**23**	**H20**	**ALD_5_Actuator**	**Pulse_(mSec.)**	**22.000000**	
**24**		**Delay_(Sec.)**		**1.000000**	
**25**		**EXPO_Actuator**	**Set_to_Open**	**0.000000**	
**26**		**MFC_Flow**	**Set_to_Value**	**100.000000**	
**27**		**Delay_(Sec.)**		**28.000000**	
**28**		**Loop_n_Times**	**Number**	**200.000000**	**LoopStrt**
**29**		**Vent**		**0.000000**	

Section S4: Computations/Simulations

COMSOL simulation of thermal transfer

Figure SI2. COMSOL thermal transfer simulation. COMSOL simulations of thermal interactions caused by conduction and blackbody radiation in the system. When the microevaporator is in contact with the substrate, there is noticeable thermal interaction between the two. When a gap is between the two, the thermal interaction is negligible since NFRHT is not included.

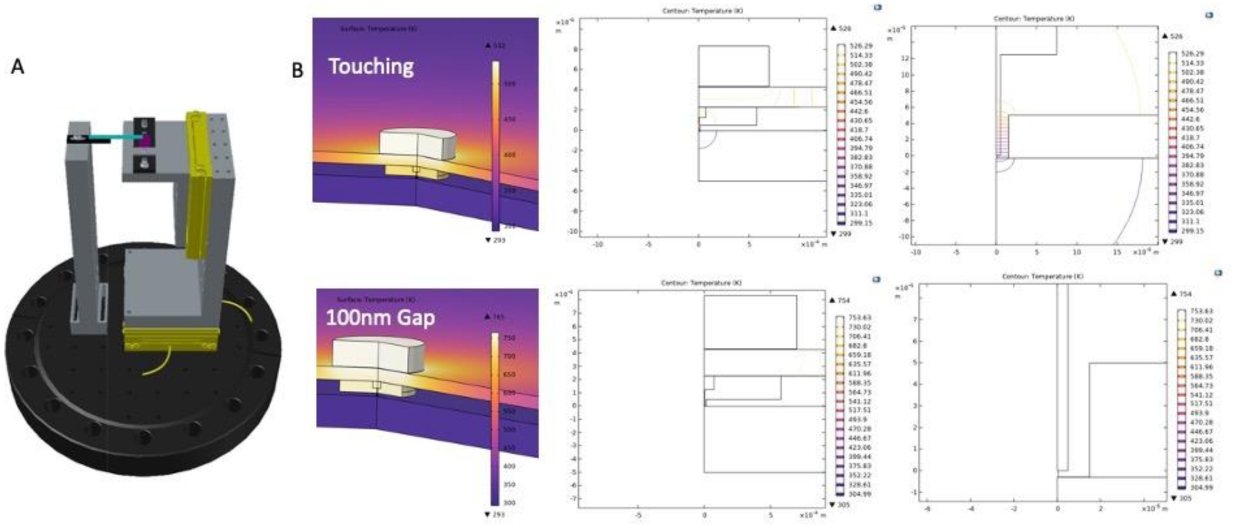

Assumptions regarding thermal interaction by conduction and blackbody radiation were verified by COMSOL simulation, summarized in **Figure SI2**. This COMSOL model predicts a 390C maximum temperature from 300mW power at the surface of the black silicon. Experimentally, imparting ~300mW of power via LED resulted in a maximum temperature of 290C. This discrepancy may be due to slightly less than 300mW being imparted due to underestimation of losses as the light passes through the glass window into the chamber and because the actual microevaporator body (1mmx2mm) is slightly larger than the 1mm diameter axially symmetric microevaporator in the model. COMSOL simulations do not show a temperature change on the microevaporator due to changes in distance, i.e. the microevaporator temperature is constant at any distance that is not in contact with the substrate.

SPARTA simulation of fluid flow through nozzle

We utilized SPARTA-DSMC to simulate the microevaporator system in order to investigate the dependence of deposition width on distance from the substrate and nozzle radius. In the model, we use gaseous Zn as the gas of choice, so the particles have a Van der Waals radius of 139 pm [47]. We also assume a temperature of 500K and a pressure of 1 x 10^{-3} Torr, and calculated the inner pressure of the microevaporator by the ideal gas law.

We chose to investigate nozzle radii of 50nm, 250nm, and 2 microns, and used a 3d simulation box to do so. Using a CAD program, we modeled the system and used SPARTA's pizza.py

toolkit to convert the surface files into SPARTA-readable surface files. To track particle collisions with the substrate, we built two substrates using the surface creation tool in pizza.py. We first used a very fine mesh, using 4000 triangles per side over a distance of 4 microns to get a particle collision resolution of 10nm by 10nm. The rest of the space was filled with a larger substrate with much less resolution to reduce computational resources – we only tracked collision onto the finer substrate using an out file that computed collisions per surface. Due to large computational power needed for this simulation, we ran this for 1 millisecond in increments of 1 microsecond and found that making increasingly fine grids near the substrate was helpful in reducing computation time. We then parsed the outfile using native Python applications to produce the graphs using MatPlotLib [48]. The resulting profiles are shown in Figure 9b.

There is also a marked agreement between our presented 3D simulations and 2D simulations of the same system, which indicates that, in general, we may be able to save on computational time by using 2D simulations instead, since they are largely without loss.

If we imagine our probability distribution for any radius *r* to be *f(r)* then for the 3D model we have:

$$[P(r \leq R) = \int_0^R 2\pi r\, f(r) dr]$$

Yielding the frequency distribution:

$$g(R) = \frac{d}{dr}\big(P(r \leq R)\big) = 2\pi R \cdot f(R)$$

In practice, we want to find a PMF (Probability Mass Function), which is done by first obtaining both distributions and then taking a sum of the form $\sum_{x=0}^{x_{max}} x \cdot n(x)$, where *n(x)* is the amount of particles *x* away from the center, to find the total weight of the distribution. We could then divide through to get a PMF.

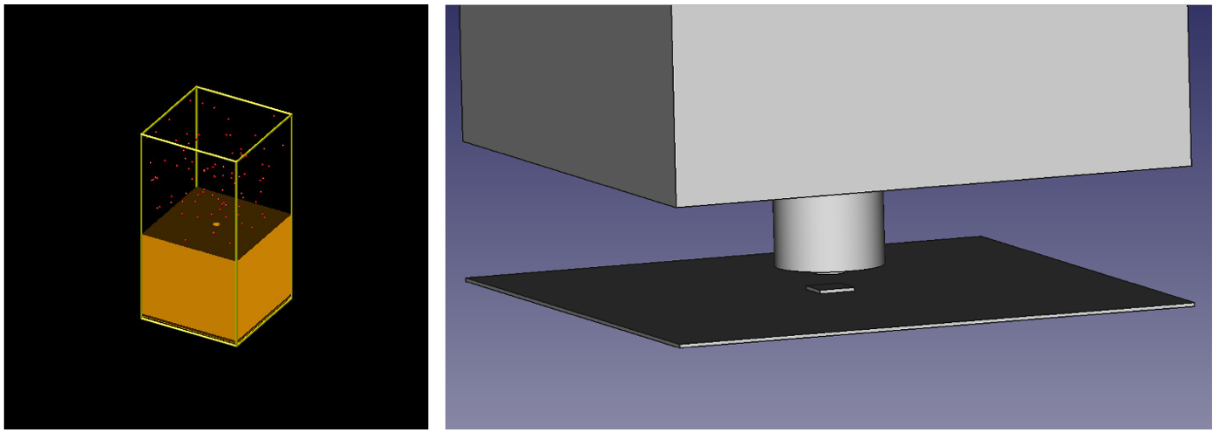

Figure SI3. DSMC 3D SYSTEM ILLUSTRATION. (left) SPARTA-generated image showing simulated system. (right) CAD file used to generate geometry in SPARTA.

Computing deposition profile from cosine law surface source equation

The deposition profiles were computed in Matlab. We start with $\frac{d\bar{M}_s}{dA_s} = \frac{\bar{M}_e(n+1)\cos^n\phi\cos\theta}{2\pi r^2}, n \geqslant 0$ (Equation 1) [44]. $\bar{M}_e$ is the total evaporated mass, r is the tangent line from the source center to the substrate, ϕ is the angle between r and the source normal, and θ is the angle between r and the substrate normal. The value of n corresponds to the directionality of the flow. n is zero for a point source that deposits equally in all directions and increases for deep, narrow evaporation crucibles that narrow the angular spread of the evaporant. $\frac{d\bar{M}_s}{dA_s}$ describes the mass deposited on a region by a surface source. As mentioned in the paper, due to the short source to substrate distance, this equation must be modified to accurately describe our system.

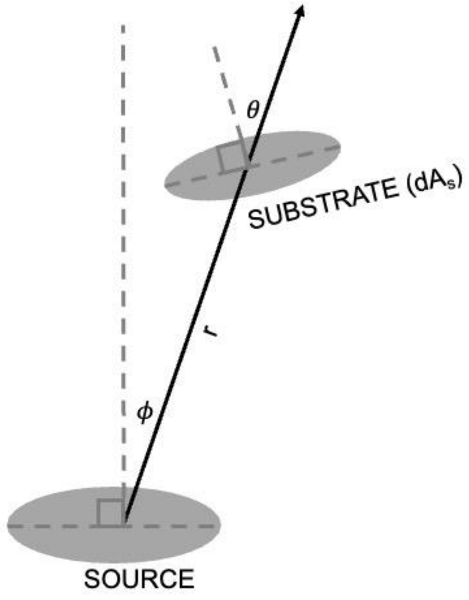

Figure SI4. Cosine law equation variables illustration. R is the line from the source to the area of interest on the substrate, ϕ is the evaporant emission angle, and θ is the receiving angle. Image inspired by [44].

To do so, we divide the deposition into 100 timesteps. We divide the nozzle diameter into equal 5nm or 50nm segments (depending on overall size), define 2001 equally spaced bins along the substrate surface, and divide the possible 180 degrees of exit angles into 10001 parts. We iterate over each timestep, and at each point along the diameter of the nozzle, at each angle of exit from said diameter point, we find a trajectory. We determine which bin along the substrate surface will be intercepted by the chosen trajectory, taking into account the height of the previous timesteps' deposited material, which allows the shadowing effect to be realized. Then, we

calculate dM using the equation, which is simplified to the form $d\overline{M}_s = \frac{A*\cos\phi\cos\theta}{r^2}$. The constant is set to A=.0000001*h*h which is scaled by deposition height since the r^{-2} contribution requires A to be different for different deposition heights, h, to result in comparable overall deposition heights for ease of viewing. At the end of each timestep, the contributions dM are added to the growing deposition.

Calculation of deposition full width at half maximum (FWHM) statistics

Height maps were collected in by scanning laser confocal microscopy (SLCM) and processed using LEXT OLS visualization software to calculate FWHM of each deposited spot. First, the maximum of the spot is found on the two-dimensional height map, then the FWHM is determined along the two perpendicular axes intersecting the maximum. FWHM along each axis is calculated as the distance between the two points of the profile which are at the height which is half of the maximum height of the deposition. The average of the two FWHM values is taken to be the FWHM of that spot. This process is repeated for every spot deposited from 5 separate depositions performed by microevaporators having 5 nozzles each. Two devices total were used, with one being used four times and the other being used once. All depositions were performed using Coumarin evaporated at 160 C microevaporator temperature, room temperature silicon substrate, 800 nm diameter nozzle diameter, and 500 nm source to substrate distance determined by thermal touchdown.
